# Manuel Pratique De La Lithotritie, Ou Lettres a Un Jeune Medecin, Sur Le Traitement De La Pierre Dans La Vessie

**Published:** 1831

**Authors:** 


### REVIEWS AND BIBLIOGRAPHICAL NOTICES.

---

Art. IX.—*Manuel-Pratique de la Lithotritie, ou Lettres a un Jeune Medecin sur le broiement de la Pierre Dans la Vessie;* par A. P. Bancal Medecin a Bordeaux. Paris: 1829. pp. 184, 8vo.

*Practical Manuel of Lithotrity, or Letters to a young Physician on breaking down the Stone in the Bladder.*

We presented to our readers, in the last No. of the Journal, some remarks on Civiale's method of curing the stone, by Dr. A. G. Smith, an enterprising physician from Kentucky, who visited Paris with the view of perfecting himself in this operation, and who intends to devote his attention, in the practice of his profession, principally to the diseases of the urinary organs. Since we shall probably have a surgeon established in the western country, well qualified to perform lithotrity, we cannot perform a more acceptable service to our readers, than to lay before them such facts as will enable them to judge of the comparative value of the operation.

M. Bancal, the author of the work which we have selected, is a surgeon of Bordeaux, whose attention having been early drawn to this subject by the high reputation which Civiale had acquired, visited Paris for the purpose of enjoying the advantage of this gentleman's instruction, and witnessing his operations. Having secured the necessary information, he returned to Bordeaux, and has since performed the operation in numerous instances, and with very considerable success. In this work he publishes the result of his experience, and illustrates the principles and success of his practice by numerous cases.

His description of the instruments used in the operation, are taken principally from Civiale's work; we shall, therefore, for the benefit of such of our readers as have not read the preceding

Nos. of the Journal, publish the description given in a review of Civiale's work on Lithotrity, in the 2d Vol. of the Western Journal of Medical and Physical Sciences.

The Apparatus consists chiefly:—

1. Of an exterior, straight metallic canula, or sheath. For an adult it should be 11 inches long. Its diameter must vary from two to four lines. It may be made of silver, gold, platina, steel, or copper—Dr. C. thinks silver the best. In operating, it is this part of the apparatus that is in contact with the urethra.

2. Of an interior tube, with one end slit down, and formed into pincers, called by Dr. C. *litholabes*. It must be longer than the other, and always made of steel. Its use is to seize and hold fast the calculus during the operation, and to withdraw the fragments when it is broken down The pincers should consist of two, three, or four branches; or rather the surgeon should be provided with several, adapted, by the number of their claws, to different cases, and to the objects to be effected. They must be elastic, so as to expand when pushed beyond the inner extremity of the exterior canula; and their ends are flattened, rounded, and incurvated. They are not exactly of the same length, so that in closing up they occupy, comparatively, but a small place, and form to the instrument a rounded head, which may be directed to any part of the surface of the bladder, with impunity to that organ. The pincers with two claws, may be used to extract small calculi from the urethra. Those with three branches, are preferred by the inventer for seizing, holding, and extracting the stone, in all ordinary cases. Those with four claws are only used under particular circumstances, which we need not enumerate. Sometimes one of the claws is moveable, and may be employed or removed at pleasure. The opposite, or outer extremity of the *litholabes* has a graduated scale which indicates the extent to which the claws are opened, and consequently the magnitude of the stone.

3. A perforating instrument, named by Dr. C., *lithotriteur*. It is this which, by a rotary or boring motion, penetrates the calculus, and effects its disintegration. It is a solid steel rod, which is introduced through the hollow shaft of the *litholabes*, or pincers. Its head is armed with teeth, or projecting points which act upon the calculus. When the pincers are shut, this jagged extremity is so enclosed as to protect the bladder from its action. The other extremity is pointed, and, near the end has a graduated scale, by which it may be accurately known how far the head of the instrument has perforated the stone. Dr. Civiale has devised two modes of augmenting the size of the aperture, made by the perforator:—1. by giving to the boring extremity, a slight degree of curvature, by which it is made to describe, in the calculus, a much larger circle,—2. by forming the head of two parts applied to each other longitudinally, and susceptible of being made to recede, so as to increase the diameter of the hole. Perforators of this kind are not so strong and solid as the others; but may be advantageously employed "when the stone is large, friable, and round."

Such are the implements which are introduced into the urethra and bladder. The remainder of the apparatus is composed of instruments, intended to connect these in their movements; to keep the pincers closed upon the calculus; to rotate the borer, and to project it forwards. Of this part of the machine it would be difficult to give an idea without the aid of the engravings, with which the book is illustrated; nor is a full description of them necessary to the object which we have in view. They consist chiefly of a turner's lathe, in steel, the beam of which is six inches long. The permanent, or fixed puppet is to be screwed to the end of the outer canula, while the moveable, clasps firmly a leaden tube, open at one end, and containing a coiled steel wire, which projects an iron cylinder against the outer end of the perforator, and imparts to it a progressive motion. On the shaft of the perforator is a pulley, round which a turner's bowstring is passed, and thus a rotary motion is imparted by the hand of the surgeon.

The hollow shaft of the pincers is prevented from passing into the bladder from the pressure of the borer against the stone, by a pressure screw, which passes through the outer tube, and by a button which is received against the external end of that canula, and which, at the same time, contributes to prevent the escape of the fluid from the bladder.

For the practice of lithrontity, our author, in his seventh chapter, has laid down minute rules, of which we shall make but a brief abstract.

The operation may be performed at all seasons of the year. The patient must be prepared for it by an attention to the means of improving his general health, if it be impaired. If this be even good, he is to be subjected to a course of mild antiphlogistics; wiih a diet calculated to prevent inflammation. So much for the preparation of his general system. The urethra must be subjected to a special treatment, calculated to diminish its sensibility, and familiarize it to the presence of instruments. For this purpose, a flexible sound must be introduced, daily, for eight days or more, and kept in the urethra for ten minutes each time. Its size should be gradually increased, until the urethra will bear, without irritation, one that is equal in diameter to the exterior canula of the boring apparatus.

The patient being thus prepared, he is placed on his back with the pelvis raised, and warm water, or mucilage is injected into the bladder, through a canula. This instrument is then to be withdrawn, and immediately afterwards, the apparatus for the operation is to be introduced, in the manner already described for introducing straight sounds. The pincers are then to be expanded, and made to enclose and secure the stone. The hollow stem of the pincers is then to be immoveably fixed to the external sheath, or canula, by a pressure-screw. The perforation may now be undertaken.

The boring may generally be continued ten minutes. On withdrawing the apparatus, the first urine that flows is colored with blood. The patient is ordered immediately afterwards to take a bath, and to observe a mild regimen. By the next day he has in general, recovered from the operation, and is ready for its repetition; to which he commonly submits with less apprehension and greater confidence than before. The number of operations must depend on the size and hardness of the stone. The fragments are either discharged with the fluid contained in the bladder, or brought away by the pincers; and great pains should be taken to ascertain that they are all evacuated."

We have lately had an opportunity of examining these instruments, and the impression made on our mind, and we believe, on the mind of every physician in this city, who saw them, was a full conviction of the practicability, and of the comparative safety and mildness, of the operation.

It is a very great mistake to suppose that the introduction of bougies in the preparation, is necessary to increase the size of the urethra. This canal, in its natural condition, is sufficiently large to admit the largest instrument used in the operation, and the only object in view is to diminish its sensibility.

The following are his directions for introducing a straight sound into the bladder, which M. B. says, is done with as much facility, as the introduction of the curved one. The urethra is divided into three portions,—from the extremity to the bulb; from this point to the prostrate gland; and from this point to the neck of the bladder. The penis being held as in the introduction of the catheter, the sound is introduced perpendicularly until it arrives at the bulb of the urethra. Here it meets with an obstruction. Retracting it a little, it is inclined at an angle of 45 degrees with the horizon, and in this direction it passes with facility until it arrives at the prostrate. Being again withdrawn a little and placed horizontally, it easily surmounts this body, unless it be very much diseased, and arrives at the neck of the bladder, when by a slight additional inclination it is easily made to pass into this viscus.

The process by which the stone is broken down, differs according to circumstances. (1) The first is by boring. The effect of this operation is well understood. The cylindrical hole formed by the instrument will be equal to the circle formed by its curved head. The result will be a fine dust which passes away with the urine, rendering it turbid. (2) When the stone has been repeatedly attacked by the foret, and its structure is very much broken, it may be crushed by pressing it against the head of the drill; and this may be frequently done if the stone be very soft, before it has been acted upon by the drill. (3) If the fragments produced by demolishing the stone, are too large to pass the urethra, a smaller instrument is introduced, the stone is seized as in the former instance, and the *lithotriteur* is worked by the hand. We will briefly notice some of the difficulties which have been urged against this operation.

1. *The pain of the operation.* It has been said that this is very considerable, and that some patients who have undergone both operations, have spoken of the pain produced by the knife, as far less than that produced by the new operation. The pain is not necessarily great, as many during the operation suffer very little, and in the interval between the sittings, often attend to their usual vocations. Much, however, will depend upon the skill of the operator, and the accidental or constitutional irritability of the urethra, and upon the morbid derangements of the urinary organs; and, consequently, it may be difficult to say *a priori* in individual cases, what the comparative amount of suffering may be. But however this balance may be adjusted, it is a fact deserving of much more consideration, that lithotrity is in every other point of view far the less formidable operation. Of the pain produced during the operation, M. B. offers the following rationale, viz: When the branches of the *litholabe* are expanded, the neck of the bladder is stretched in a triangular form, and in the changes in the position of the instrument which are necessarily made during the search for the stone, great irritation may occasionally ensue. Hence it is in the urethra and the neck of the bladder that the injurious effects of the operation are principally produced.

2. The second unpleasant consequence of this operation is, a *swelling of the testicle.* M. B. states that this affection frequently results from the use of bougies in the cure of strictures of the urethra. Whatever may be the explanation, it is no doubt analogous to the transfer of irritation from the urethra to the testicle, which takes place in gonorrhea.

It has been alledged by some, that *the bladder is liable to be siezed* between the branches of the litholabe. This M. B. says neither he, nor Civiale, has ever known to take place; and indeed, from the structure of the instrument, he considers the accident to be impossible. When fungous growths, however, spring from the mucous membrane of the bladder, these may be seized, and serious consequences may, possibly, result.

4. *Symptomatic fever* occasionally results from the irritation of the urethra, of so serious a nature as to require the operation to be suspended, and sometimes to be given up entirely.

5. *Inflammation of the bladder* has been urged by some as a frequent consequence of the operation, and it is stated in a late number of the Medico Chirurgical Review, that a stage of depression sometimes follows the operation, and that the patient sinks without reaction ever taking place. The first of these affections M. B. thinks is not likely to take place, and as he has made no mention of the other, it probably very seldom occurs.

The author has adopted the plan, of illustrating the nature of the operation and of the difficulties to be met with, by a detail of cases: we shall pursue the same course, believing that thus our readers will be better enabled to judge of the true value of the operation, and to determine how far the objections which have been made are justly chargeable to it.

The division of his cases is the same as that given by M. Civiale, for which we shall again refer to a former No. of this Journal.

'" In the first series," says he, " I shall present only the cases of patients whose conditions were most favorable to the operation.

'" I shall refer to the second, those in which the disease was far advanced, and offer complications which rendered the operation more difficult.

'" Finally, in the third, I shall relate cases in which a successful resort to lithontrity was impossible." ' p. 76.

" The cases recorded in the first series, are not exactly similar; but in all of them the calculi had not attained to any great magnitude, or were few in number; the bladder was nearly sound; the urethra and prostrate gland were healthy and well formed, and the general health of the patient was good."

"Cases referred to the 2d and 3d of these series, do not of course resemble each other so closely as the first; seeing that the circumstances which render the operation difficult or impracticable, are necessarily much diversified.

Strictures of the urethra, and excessive sensibility of that tube; a similar state of the vital properties of the bladder; chronic cystitis, organic changes in the parieties; diseases of the prostrate; the multiplication of calculi; and a bad state of the general health of the patient, are circumstances that render the operation of lithontrity difficult and dangerous. It should be regarded as impracticable or as contra-indicated;—when the disease has lasted for so long a time that the calculus has acquired great dimensions, and the bladder, contracting firmly upon it, has become horny; when the calculi are numerous and of considerable size, and the health of the bladder, and that of the general system is bad; generally, when the stone is encysted; when it is large, and lodged in the urethra; when that tube from *organic* changes has become, in a great degree, impervious; when the nucleus of the stone, is a foreign body of such a kind, that it cannot be disintegrated; finally when the kidneys are in a state of organic disease. Many of these conditions render lithotomy not less than lithontrity, hazardous, impossible, or fatal; Dr. C. has, however, reported some cases, in which, after a reluctant trial of his method, he was induced to desist, and the patients were afterwards cured by the old operation."

Case 2. M. Dupuis, of Bordeaux, aged 66 years, of good constitution, and exempt from the crowd of infirmities which attend old age, presented himself for advice. Six years since, he began to pass small spherical bodies from the bladder, of the size of small peas; and three years ago, he complained of unequivocal symptoms of the stone. He was not sounded, however, but contented himself with pursuing a regimen, and avoiding every cause which could aggravate his sufferings.

In August, 1828, he applied for the assistance of M. Bancal. Having explored the bladder and ascertained the presence of several calculi, he commenced the preparation, and proceeded to operate on the 16th of September. The instrument was introduced with facility, notwithstanding a considerable enlargement of the prostrate, a stone was seized and ground down, and several fragments crushed between the branches of the instrument and the head of the foret. After this sitting, which lasted fifteen minutes, the urine was voided slightly bloody, and containing numerous fragments, some of the size of a small lentile. He experienced no inconvenience but a slight pain in the neck of the bladder, and the canal of the uretha, which, however, did not prevent him from rising early on the following day and taking a morning walk.

The operation was again repeated on the 20th of this month. On this occasion the difficulties encountered led M. B. to suspect a fungous growth from the bladder. He met also with other impediments from the displacement of the bladder, in consequence of the accumulation of feces in the rectum; a lavement not having been administered preparatory to the operation, in conformity with his directions. Several fragments of the stone were seized and operated on, but with great difficulty in consequence of these circumstances. The retraction of the instrument was also attended with some difficulty, in consequence of one of the branches of the *litholabe* becoming entangled with this fungous growth.

This sitting lasted twenty-five minutes, and was very painful and fatiguing to the patient, who fainted soon after, probably from having neglected to take nourishment before the operation. On taking some refreshment, he revived so far as to be able to void his urine, which was bloody, and contained much of the detritus of the stone. A warm bath, fomentations, and emollient drinks were recommended. A chill followed, succeeded by fever, which continued through the night. The emission of urine was accompanied with sharp pain in the neck of the bladder, and the uretha. The antiphlogistic regimen, kept up for three days, calmed the local irritation, and reduced the parts to their healthy condition.

After this operation, he passed several fragments, one of which weighed 9 grs. and 12 oz. Fifteen days after the operation, he expelled four more, which he did not preserve, but which, from the pain attending their expulsion, he supposed to be larger than the preceding.

The condition of M. Dupuis was now very much improved, almost all the symptoms of the stone having disappeared, when he was suddenly seized with a rheumatic affection of the lower extremities.

On the 2d of November, M. D. being cured of his rheumatic affection, the instrument was again introduced, the extremity of which came again in contact with the substance before mentioned, which was supposed to be an excresence from the surface of the bladder, and several physicians present satisfied themselves of the truth of this opinion. The sound being withdrawn, a lithontripteur was introduced, but no calculus could be found.

After this examination, he descended from his apartment and dined with his family, and still enjoys good health, and attends to his business.

This case shows the importance, 1. Of evacuating the contents of the rectum before commencing the operation; and since this time M. B. always retracts the instruments, when he ascertains that this has not been done. 2. It proves that the presence of excrescences from the mucous coat of the bladder does not always prevent the success of the operation. 3. Great age is no obstacle to the success of the lithontripteur, provided serious organic derangements of the urinary organs do not accompany the disease which calls for the operation.

Case 3. M. Rollan, of Nerac, aged 32, of large stature, good constitution, and sanguine temperament, experienced two years since those symptoms which indicate the stone. In the month of September, 1827. he came to Toulouse, and placed himself under the care of Dr. Viguerie, who sounded the patient, and ascertained the existence of a stone, and of a considerable enlargement of the prostrate.

In October he came to Bordeaux to claim the assistance of M. B. and submit to lithontrity. He suffered much after voiding his urine; and on leaving the urine to stand, a copious deposit took place. The existence of the stone having been confirmed by the sound, he was subjected to two sittings. After these operations he voided a great deal of detritus, but the bladder being very irritable, the second operation brought on an ischury. Anxious to return home to attend to his business, he set out from Bordeaux in company with a physician, and arrived at Nerac without any accident.

Some months afterward, he was cut for the stone, by M. V. At the first view, this surgeon was surprised to find no mark of the lithontriptor, on the calculus, but on scaling it with the edge of a scalpel, there came from the cylindrical cavity made by the foret, a round plug which had completely filled it, and which was doubtless composed of uric acid.

This fact appears to shew that the elementary principles of the calculus, with which the urine was saturated, had a greater affinity for the surface of the cavity made by the instrument, than for the spherical surface. What law of molcular attraction presides over this phenomenon, M. B. does not attempt to determine.

M. B. takes this opportunity to call the attention of his correspondent to another important fact, which had been previously noticed by Deschamps. The sound, when introduced into the bladder in this instance, gave no distinct sensation of collision against a hard body, and it was the same case with the expanded branches of the *litholabe.* This he attributes to the abundant secretion of mucus covering the stone, and preventing the sound which usually follows from the collision of the instrument, and distinctly ascertains the presence of the stone.

Case 4. This case is intended to shew that when the urinary organs are very much diseased, when the capacity of the bladder is diminished by thickening of its coats, when it voids great quantities of mucus mixed with pus, and when the prostrate gland is very much enlarged; under these circumstances, although we may succeed in destroying the calculus, the patient will remain in a state of very troublesome disease and infirmity.

Jean Leglise, aged 72, an agriculturist from the vicinity of Bordeaux, began to experience the symptoms of the stone about four years since. This patient, having read the works of Desault, had taken such a prejudice against lithotomy, that he said he would rather die than submit to it.

He came to consult M. B., in Dec., 1828. His constitution, originally good, was very much impaired, and his sufferings were very great. He was obliged almost every moment to attend to the call to evacuate his urine, which came away in small quantity, and deposited a great quantity of very thick mucus, mixed with pus, and exhaling a very decided ammoniacal odour; he often had fever; enjoyed little sleep; his appetite had failed; and his digestion was bad. He had also a large inguinal hernia on each side.

M. B. refused to operate on this man, and proposed lithotomy. To this he refused to submit, and pressed his entreaties with so much solicitude, that M. B. at length consented to resort to the new operation.

After the ordinary preparation which presented nothing remarkable, he proceeded to the operation in January, and destroyed the stone at four sittings.

At each application the stone was seized, but it was proved to the satisfaction of the physicians present, that the bladder was very much indurated, by the small amount of injection which it received.

After the operation, the urine continued to deposite great quantities of mucus, loaded with pus, and to require to be frequently voided. The constitution remained weak, although the painful symptoms arising from the presence of the stone were obviated; and the patient expressed himself much gratified with the success of the operation. A month after the operation he came on foot during a season of severe cold, when the ground was covered with snow, to see M. B., and for the two years that he lived after this, he was able to attend to his business, using only the precaution very frequently to void his urine, which continued of the character above described.

Case 5. M. S., a surgeon, a resident of Bazas, aged 55, of a temperament sanguine and nervous, had suffered several years from a stone, the existence of which was ascertained by the sound. In March, 1827, he came to Bordeaux, to place himself under the care of M. Bancal. From Bazas to Alangon, whither the patient went to take the steam boat, the road is paved, and the jolting of the carriage brought on severe pains in the bladder, and bloody urine. After his arrival in Bordeaux, M. S. had fever; after taking the rest necessary to his condition, he was sounded, and underwent the preparatory treatment. The bladder and urethra being very irritable, M. B. commenced with the introduction of a very small sound, which was followed by symptomatic fever. After the preparation had continued 12 days, M. S., accustomed to a country life, became impatient for the operation, and M. B. reluctantly consented to attempt it. The bladder could not be distended by the injection. The lithontripteur was introduced with facility, but at the moment when it passed the neck, the patient complained of severe pain, and the branches of the *litholabe*, when expanded, produced excrutiating pain. The parietes of the bladder were very much thickened, and, being unable to move the instrument without producing great suffering, M. B. gave up the attempt. M. S., despairing of relief, returned home the next day.

M. B. remarks that this patient was in a condition favorable for lithotrity before his journey. He enjoyed good health, had a good appetite, and suffered little from the stone; but he was naturally very irritable, and his journey produced a state of high irritation in the urinary organs. Persons afflicted with calculi, often travel with impunity, but no one can determine with certainty, *a priori*, the irritability of the bladder which contains a stone. Those who seem to be affected but little by its presence, often suffer very severely when they attempt to travel, and should expose themselves with caution from the dangers of being caught in situations, where the means of relief are not likely to be met with. The necessity of attending to this circumstance, will appear from the following case, which shews, in a striking manner, the danger to which patients affected with calculi are exposed from long journeys.

Case 6. M. Eusebi, *conservateur* of music and painting to the king of Spain, aged 56, of large stature, and of a strong constitution, had been affected with gravel 30 or 40 years. Two years since, he began to experience the symptoms of the stone, and his condition becoming progressively worse, he determined to pass into France to undergo Civiale's operation, which had not then been practised, and is still little known in Spain. The journey from Madrid to Bayonne, occupied 40 days. Journeying by small stages, he was often compelled to stop, to subdue the irritation, which travelling produced, and which rendered his journey tedious, painful, and perilous.

At Bayonne, the catheter was introduced for ischuria, by a surgeon, who, without entering the bladder, made a false passage. This accident was accompanied by a severe hemorrhage, and produced violent pain and convulsions. After the disappearance of these symptoms, he was carried in a boat to Mont de Marsan, from thence in a litter to Langon, and thence in a steam boat to Bordeaux.

After 15 or 20 days of repose, M. B. commenced the preparatory treatment. The prostrate was very much diseased, and he suffered very much from the presence of the sound in the bladder, especially when it caused any displacement of the stone. The introduction of the instrument always produced a temporary flow of blood; bougies were introduced with facility, but as soon as they passed the neck of the bladder, he perceived a sensation which informed him of the effusion of blood, combined with a feeling of heat, and exclaimed, "You make my urine bloody." M. B. supposes that the vessels of the prostrate were varicose, and were ruptured by the passage of the instrument.

In March, he was subjected to his first sitting. After injecting an emollient fluid, the lithontriptor was easily introduced, and its extremity gave the sensation of a soft body in the base of the bladder, of the nature of which M. B. could form no idea. He displaced the stone which was situated in the superior part of the bladder, and succeeded in grasping it, and operating upon it in three different positions. M. S. suffered much, and voided bloody urine with much detritus. The symptoms of local irritation which succeeded were calmed by an appropriate treatment.

On the 10th day, when M. B. was prepared to repeat the operation, the patient was attacked with a violent sciatic pain in the right side. This pain, of the nature of rheumatism, extended first to the leg, and then to the shoulder. This affection yielded to appropriate treatment, but as the rheumatic pain gave way, the bladder became so much diseased that it was impossible to repeat the operation.

The patient, very impatient, and opposed to the temporizing practice which his condition required, set out for Paris, to place himself under the care of Civiale, and embarked at Bordeaux, to go by sea to Havre.

Before his departure from Madrid he was no doubt in a condition favorable for lithotrity. The volume of the stone was not too great. The diameter of the urethra was sufficiently large for the introduction of the largest instrument. The morbid condition of the bladder was occasioned by the journey, and by the false route made by the catheter. M. B. also thinks that these symptoms were aggravated by the metastasis of a constitutional rheumatism to which the patient was liable; and he asserts that he has met with other cases in which a similar circumstance has occurred, so that he is always on his guard where he has reason to apprehend a similar accident.

This patient arrived in Paris about the middle of August, and died a few days after. Dubois, Marjolan, and Civiale, who were called upon to visit him, reported him to be affected with severe organic lesions, and that his bladder was very much thickened.

Case 7. Orignac, of the department of Gironde, aged 32 years, of a good constitution, presented the symptoms which designate the presence of the stone in the bladder. In April, 1828, he placed himself under the care of M. B., who, having explored the bladder, found it very little diseased and of a large capacity. After several days preparation, the patient fixed his residence near M. B., to be convenient for the operation. The operator could neither seize nor displace the stone, which remained constantly in the right side of the posterior part of the bladder. The position of the patient was changed, and part of the fluid evacuated, but nevertheless, although all the physicians present assured themselves of the presence of the stone, it was impossible to seize it. This patient shortly after returning home, M. B. was deprived of the opportunity of making another attempt. He attributes the difficulty of seizing the stone in this instance, to its flattened form. Although he does not think with Civiale, that the general form of the calculus can be ascertained, he thinks himself justifiable in drawing this conclusion in this particular case.

☞ The cases which have been hitherto reported, have been those in which, although complicated, the new operation might be employed with success. The following are instances in which it could not be employed.

Case 8. M. Delerm, a notary from Paillet, near Bordeaux, aged 70 years, of a good and strong constitution, has been subject to the gravel about 30 years. About five years since he began to experience the symptoms of the stone. He was subject to wandering rheumatic pains which several times have been complicated with the peculiar symptoms of the stone. In the spring of 1828, he came to consult M. B., who, by introducing a sound, ascertained the presence of the calculus. On the 17th of March he commenced the preparation, by introducing a bougie of a large size, which was continued until it could be introduced without producing irritation. On the 21st, he proceeded to the operation, introduced the instrument with facility, and having seized the calculus, ascertained the existence of another, by the shock which he perceived on moving the instrument. The sitting lasted but five minutes, and was followed by no remarkable accident.

On the 26th, he again submitted to the operation, and after having taken the bath, he dined immediately, and received the visits of his friends. The operation was repeated a third time; but on the day fixed for the 4th operation, he experienced a desire of voiding his urine more frequently, accompanied with dull pains in the bladder. On the night of the 5th or 6th of April this condition was aggravated, the hypogastric region became painful, fever was excited, the urine was voided with difficulty, and contained a great quantity of mucus; he perceived a weight in the return with tenesmus, his pulse manifested a febrile rhythm. A rigorous antiphlogistic treatment was opposed to this condition; bleeding general and local, warm baths, mucilaginous drinks with nitre, narcotic enemata, with low diet, and rest. The general irritation calmed itself, but the local continued and kept the patient in the most severe pain. Venice turpentine, balsam copaiva, and anodynes did not remove the disease. The acetate of morphine, administered in small doses, produced such a determination to the head that it was necessarily discontinued. Injections into the bladder, of decoction of mallows and poppy heads, although managed with care, produced a fever which lasted four days. Finding all medical treatment ineffectual, M. B., after six weeks, proposed lithotomy, to which the patient submitted.

M. B. thinks that this state of excessive irritation was not produced by the operation, and offers the following reasons.

The operation had been twice performed without any accident; and the third operation was attended with less inconvenience than the preceding; six days passed without any increase of the symptoms, a length of time which was certainly not required for the manifestation of injury produced by the irritation of so delicate an organ. He thinks it was a metastasis of a rheumatic affection to which the patient was subject, and which being suddenly turned upon the bladder, exasperated the symptoms of the disease already existing there. The patient had frequently before been subject to violent pains in the bladder, sometimes placing his life in extreme jeopardy, and which his physician regarded as a transfer of rheumatism to this organ. The sufferings of the patient being unmitigated, he was cut by the bilateral operation on the 6th of July, and recovered without any accident.*

Case 9. M. Bernard Hugot, of the department of Gironde, aged 65, of a good constitution, and corpulent habit, came to Bordeaux in July, 1827, to undergo an operation for the stone, from which he had been suffering for several years. The diseased condition of this patient, his advanced age, the severe symptoms which manifested themselves in his urinary organs, prevented M. B. from expecting any thing from lithotrity. The

* M. B. attributes the happy result of the operation by the knife in this instance, to the good moral and physical constitution of the patient. One circumstance worthy of remark with respect to these calculi is, that although three large calculi were removed, their surfaces were so rough, that when shown to M. Dubois, he said that if he had extracted one of them from the bladder, he would scarcely have thought of searching for another.

first sitting produced such a degree of irritation, that he gave up the idea of lithotrity, and performed the bilateral operation; Fifty-five calculi, the largest the size of almonds, the smallest, of beans, were taken from the bladder, when the patient recovered without any unfavorable symptoms.

Case 10. In this case, M. B. having failed in the new mode of operating, attempted to extract the stone, but failed in consequence of its being encysted in a fold of the bladder. The patient survived the operation, however.

Case 11. The patient was a merchant of Bordeaux, between 50 and 55 years of age, of small stature. He had been subject to the gravel nearly 40 years, and for ten years had had symptoms of the stone; having frequent paroxysms, which kept him from 5 to 20 or 30 days in a state of continual fever, and intense local suffering. The great size of the stone made M. B. unwilling to attempt lithotrity; nevertheless, as the patient would hear of no other operation, he commenced the preparation at a very rigorous season of the year. A sound of the very smallest size, No. 4,* was first introduced; on the second day, No. 5, and on the third day the introdnction of No. 6 produced a slight degree of fever. At this period the thermometer stood at 6 degrees above zero of Reaumur, and M. B. remarks that severe cold has a very marked influence upon diseases of the bladder. For eight days after this, he enjoyed perfect freedom from fever, when he suddenly began to experience its precursors, and immediately the most violent febrile symptoms made their appearance. The local symptoms were much aggravated; the abdomen swelled, vomit and hiccough supervened, and the patient died in a few days.

The death of this man was attributed to the operation; but we think, with M. Bancal, without reason. The lithontriteur had never been used, and the sound was introduced with facility and without producing the least pain. The medical treatment of any patient is necessarily very much withdrawn from the public eye, and, in addition to this, few possess the qualifi-

* No. 9 is a sound of three lines in diameter, and they increase or decrease a fourth of a line with each number.

cations, or are willing to take the trouble, to estimate the value of a remedy by any other criterion than that of its success. It is therefore not to be expected, that when a patient dies, who is subjected to a new mode of treatment, the public will take into consideration the adventitious circumstances, whch may have influenced the fatal event.

*On the operation of Lithotrity in the Female.*

It would, at first view, be supposed that lithotrity was more easy in the female than in the male; but experience proves the contrary, and M. B. offers the following explanation of the fact.

The bladder rests upon the uterus in such a manner, that the transverse diameter is greater than that from before backward, even when it is distended; and when the patient is placed in the position for the operation, the urethra opens into the very lowest part of the bladder, and, of consequence, into the place where the stone is usually met with. The urethra being short, straight, and distensible, admits the introduction of the instrument with comparative facility; but when the instrument is introduced, it passes the stone before the branches expand, and it is afterward very difficult to grasp it; indeed it is frequently impossible to do this without changing the place of the tone, by altering the position of the patient. Passing over one successful case, we shall give one or two cases, which terminated more unhappily.

Case 2. Madame M., of Bordeaux, aged 60, of good constition, corpulent, suffered some time with pain in the urethra after evacuating the urine. M. B. was consulted, and having ascertained the presence of a stone, proceeded immediately to the operation; but found it impossible, either at this time or on another occasion, to seize the stone. She experienced so little inconvenience from the search, that after the bath, she proceeded immediately to her customary domestic avocations. M. B. referred the difficulty to the following circumstances:

1. The relaxation of the abdominal parietes had permitted the intestines to fall forward, and carried the bladder, which was very large, in the same direction, placing it in a direction almost perpendicular to the urethra.

2. The extremity of the instrument, when introduced into the bladder, came immediately in contact with a soft ovoid tumor, formed by the uterus, which rendered the antero-posterior diameter of the bladder very short, so that it was impossible for the branches of the *litholabe* to expand without depressing the extremity of the instrument very much between the legs of the patient.

3. The calculus kept itself constantly out of the reach of the branches of the instrument, and was concealed in the recesses formed on each side of the uterus.

It was the intention of M. B., if another attempt at lithotrity should prove unsuccessful, to resort to lithotomy.

Case 3. *In which the operation was contraindicated.* Madame T., of Bordeaux, aged between 75 and 80, of good constitution, inclined to corpulency, had been suffering from the stone, for several years, and had discharged several small calculi before M. B. saw her. He was consulted by her in January, 1827, and on sounding her, found a large stone immediately at the neck of the bladder. After the ordinary preparation, the patient was subjected to the operation, and the stone was, with considerable difficulty, seized; but its great size prevented the *litholabe* from taking hold of it with firmness sufficient to bear the action of the drill. The consequences of this operation were troublsome and painful, but yielded to an appropriate treatment. M. B. having desired a consultation, it was decided by the physicians who met him upon that occasion, that the patient was affected with an herpetic affection, involving the genito-urinary organs, and that this affection probably increased the abnormal irritability of the urethra and bladder; but that since the obstinacy of the patient prevented a resort to lithotomy, the only other alternative should again be tried.

The resort to lithotrity proved ineffectual, it being impossible to secure the stone within the grasp of the instrument. The symptoms consequent on this operation were calmed in a few days; but the patient fell a victim, shortly after, to a fever, complicated with a severe affection of the organs of respiration. We will give the account of the *post mortem* examination, as well to exhibit the great extent to which disorganization of the different parts of the system may take place without immediately destroying life, as to illustrate a remark which we presently intend to make.

The lungs were emphysematous, and their parenchyma scattered with calculous granulations; the ventricles of the heart were enlarged; the internal membrane of the stomach in a state of inflammation—the pylorus schirrous; the left kidney was in a state of softening, (*ramollissement*,) and the right, of suppuration; the ovaries were perfectly disorganized; the uterus and vagina bore traces of inflammation, as did also the rectum, which was closed by a stricture, to such an extent as scarcely to admit a writing quill; the walls of the bladder were thickened and hardened; and the calculus which it contained was 32 lines long, 23 lines broad, and 6 lines thick.

The cases which we have published, admitting them to be fairly reported, will bring most of our readers to the conclusion, that the morbid derangements which prevent the application of Civiale's instruments, are not so exclusive as is usually supposed, and consequently, that the operation is applicable to a much greater variety of cases than public opinion now assigns to it.

The irritation produced by the introduction of the instrument, it is evident, must depend very much upon the skill of the operator. It would not be expected that a bladder, which had long been subjected to the irritation arising from the presence of a stone, would suffer very materially from the introduction of this instrument, if guided by a skillful and careful hand; and, accordingly, the irritation, M. B. states, is generally in the urethra; and, indeed, we might suppose, from what we observe of the symptoms of the stone, that irritation, even if produced in the bladder, would manifest itself in the canal.

One other remark upon this subject we deem it very important to make. The operation of lithotrity, and the preparatory treatment, sometimes occupy a very considerable period. During this period, whatever disease may befall the urinary organs, will be laid to the charge of this operation. When we consider that these organs are the seat of a very considerable irritation, and from this circumstance, from many constitutional diseases, we alone see to how many unfounded imputations this operation will be subjected. All do not die from lithotrity, whose existence is terminated while under the care of the operator; nor are all the diseases of the urinary organs, which arise during the process of the cure, to be attributed to this operation.

The friends of this operation have never claimed it to be universally applicable, and are willing to acknowledge, that while in favorable cases it is far more mild and safe than the knife, there are cases in which the latter may be very successfully applied, where the former could not be used without subjecting the patient to infinitely greater pain and danger. Indeed, the history of one or two of the cases above reported would appear to prove, that such a degree of morbid sensibility as would preclude lithotrity, may be no obstacle to the reunion of the parts, when cut by the gorget.

We have no expectation of seeing this operation entirely supplant the gorget; but if we may venture to express the confidence derived from the inspection of the instruments, from the favorable reports of others, and inspired by the enthusiasm of the enterprising gentleman who proposes to operate in this country, we feel confident that, in this country, it will be found applicable to a large and increasing proportion of cases. We are anxious to see a qualified operator in the West. We are rapidly becoming the rivals of the East, in many respects; and we are anxious to rival the Tramontane Region in every thing. In this view, we have claims upon the services of Dr. Smith, which we hope he will not refuse to acknowledge.

As the works of Dr. Darwin are very little read, at present, it may not be uninteresting to our readers to be informed, that an operation of this nature was a subject upon which he reflected much, and that he has given an outline for the construction of the necessary instruments, by which an inventive mechanic might easily have arrived at the instrument of Dr. Civiale. Though the extract is rather long, we will venture to give it as a matter of literary curiosity.

"A curious account is given in a letter to Sir John Sinclair from Colonel Martin; who asserts, that, after using bougies and injections into the bladder, the passage of the urethra became less sensible to pain, and he was enabled to introduce small files (I suppose, with their backs smooth) and that by these he gradually filed away the stone, as it lay in the neck of the bladder. When the stone did not properly present itself, he introduced warm water by injection into the bladder, and thus, by again endeavoring to discharge it, brought forward the stone to the neck of it. He used the file three times in twenty-four hours from April till October. Medical Journal, No II. p. 121. If this process should be again attempted, perhaps the file might be introduced through a flexible canula, with a metalic hood at the internal end of the canula to cover the back of the file, so as to prevent the friction of it against the urethra, or neck of the bladder. If the urethra, by frequent trials, should become so insensible as to admit easily the introduction of a metalic canula, might not two fine steel wires properly tempered be joined at one end by a hinge, and thus introduced through the canula into the bladder; and when protruded beyond the extremity of the canula, they might open by their elasticity so as to receive the stone, and confine it against the end of the canula, by retracting them? The proper direction of the wire-springs, so as to open when they are pushed through the canula, must be previously given them. If this could be managed, a small file or borer might at the same time be introduced through the canula, the handles of which might consist of joints to permit them to bend in all directions, and thus the stone might be broken to pieces by a few trials; or if it was a soft or fragile stone, the retraction of the wire-bow might divide it at every trial, till it became almost reduced to powder. A little mechanical ingenuity might be necessary in the construction and use of this machinery; but I believe it not to be impracticable, since I read the above account of Colonel Martin, though I had often before thought of it with despair of its successful application." C.

